# Stages of preadipocyte differentiation: biomarkers and pathways for extracellular structural remodeling

**DOI:** 10.1186/s41065-022-00261-w

**Published:** 2022-12-27

**Authors:** Zhihan Hu, Yi Liu, Zongjiang Yao, Liming Chen, Gang Wang, Xiaohui Liu, Yafei Tian, Guangtong Cao

**Affiliations:** 1 Department of Clinical Medicine, Ningxia Medical University, Yinchuan, 750000 China; 2 Department of Burn Plastic Surgery and Wound Repair, Second Hospital of Lanzhou University, Lanzhou, 730030 China

**Keywords:** Preadipocytes, Adipogenic differentiation, Angiogenesis, Extracellular matrix, Fat graft, Simpson–Golabi–Behmel syndrome

## Abstract

**Background:**

This study utilized bioinformatics to analyze the underlying biological mechanisms involved in adipogenic differentiation, synthesis of the extracellular matrix (ECM), and angiogenesis during preadipocyte differentiation in human Simpson–Golabi–Behmel syndrome at different time points and identify targets that can potentially improve fat graft survival.

**Results:**

We analyzed two expression profiles from the Gene Expression Omnibus and identified differentially expressed genes (DEGs) at six different time points after the initiation of preadipocyte differentiation. Related pathways were identified using Gene Ontology/Kyoto Encyclopedia of Genes and Genomes analyses and Gene Set Enrichment Analysis (GSEA). We further constructed a protein–protein interaction (PPI) network and its central genes. The results showed that upregulated DEGs were involved in cell differentiation, lipid metabolism, and other cellular activities, while downregulated DEGs were associated with angiogenesis and development, ECM tissue synthesis, and intercellular and intertissue adhesion. GSEA provided a more comprehensive basis, including participation in and positive regulation of key pathways of cell metabolic differentiation, such as the “peroxisome proliferator-activated receptor signaling pathway” and the “adenylate-activated protein kinase signaling pathway,” a key pathway that negatively regulates pro-angiogenic development, ECM synthesis, and adhesion.

**Conclusions:**

We identified the top 20 hub genes in the PPI network, including genes involved in cell differentiation, ECM synthesis, and angiogenesis development, providing potential targets to improve the long-term survival rate of fat grafts. Additionally, we identified drugs that may interact with these targets to potentially improve fat graft survival.

## Background

Fat grafting is widely used in plastic surgery to restore soft tissue defects. However, fat transplantation poses several challenges, such as high absorption rates and low long-term survival rates. The "graft replacement theory" explains why fat grafting can be as high as 90% resorbable. In vivo experiments in animals demonstrated that the vast majority of fat cells in the graft had died on the first day due to hypoxia [1]. Only some cells near the graft edge of the donor site survived, but preadipocytes survived up to 72 h. Proliferation of surviving cells was observed on the third day, and the area of proliferating cells showed a significant increase on the seventh day, indicating that regeneration of the graft had begun. This suggests that the death of adipocytes does not imply necrosis of the graft tissue but can trigger a regenerative mechanism, namely the adipogenic differentiation of preadipocytes in the graft [2, 3]. Animal experiments have also demonstrated that preadipocytes differentiate into vascular endothelial cells with pericyte shape [4, 5] and express pericyte marker proteins. Pericytes induce the appearance of vascular endothelial progenitor cells, suggesting that precursor adipocytes can promote revascularization of grafts through different directions of differentiation [6]. Therefore, it is essential to explore the hitherto obscure mechanisms of preadipocyte differentiation and how they form connections with the surrounding stromal tissue.

Preadipocytes from multiple species have been studied extensively; however, these cells have a limited lifespan, and primary human preadipocytes rapidly lose their differentiation when proliferating in vitro. The study of human preadipocytes is hindered by the tissue origin and variability among different adipose tissues. Human Simpson–Golabi–Behmel syndrome (SGBS) preadipocytes, human diploid cells extracted from the adipose tissue of patients with SGBS, provide a more reliable tool [7–10], as they are neither transformed nor immortalized, differentiate in chemically defined serum-free medium, and can achieve > 90% adipogenic differentiation by passage 50. At high rates, these cells retain their adipogenic differentiation capacity even up to 50 passages. The SGBS model has been used in several studies, including pharmacological testing, genetics, and characterization of adipogenesis and adipokines [7, 11–13]. However, the influence of biomarkers and pathways during SGBS preadipocyte differentiation on the biological behavior of grafts displayed in the recipient area after fat transplantation has not been investigated. Exploration of potential drugs that could reverse the generally lower retention rates after fat grafting is also a key area of research. With the rapid development and widespread application of high-throughput system technology, bioinformatics analysis can serve as an effective tool for exploration in this direction.

In this study, differentially expressed genes (DEGs) were analyzed at seven time points before and after adipogenic differentiation of SGBS cells using two SGBS cell differentiation-related gene chips downloaded from the Gene Expression Omnibus (GEO) database. The biological mechanisms at different time points of differentiation were investigated using Gene Ontology (GO), Kyoto Encyclopedia of Genes and Genomes (KEGG), and Gene Set Enrichment Analysis (GSEA). Furthermore, a protein–protein interaction (PPI) network was established using cytoHubba to identify the hub genes of multiple biological pathways. Potential drugs that may interact with the hub genes were also analyzed.

## Results

**Fig. 1:**
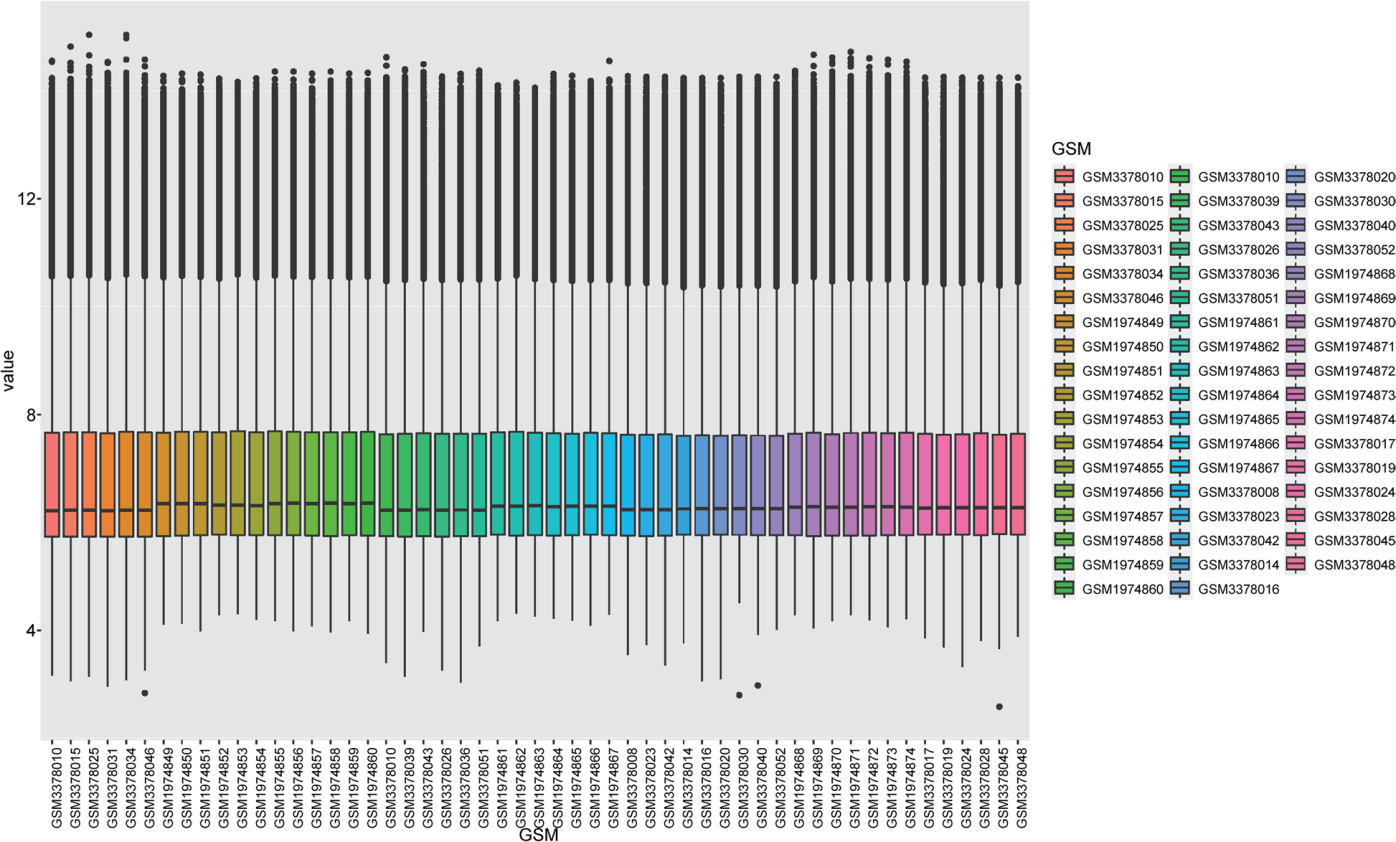
A boxplot was created for two datasets after normalization

We normalized the merged dataset and plotted boxplots (Fig. 1).

### Identification of DEGs at different time points of differentiation

**Fig. 2:**
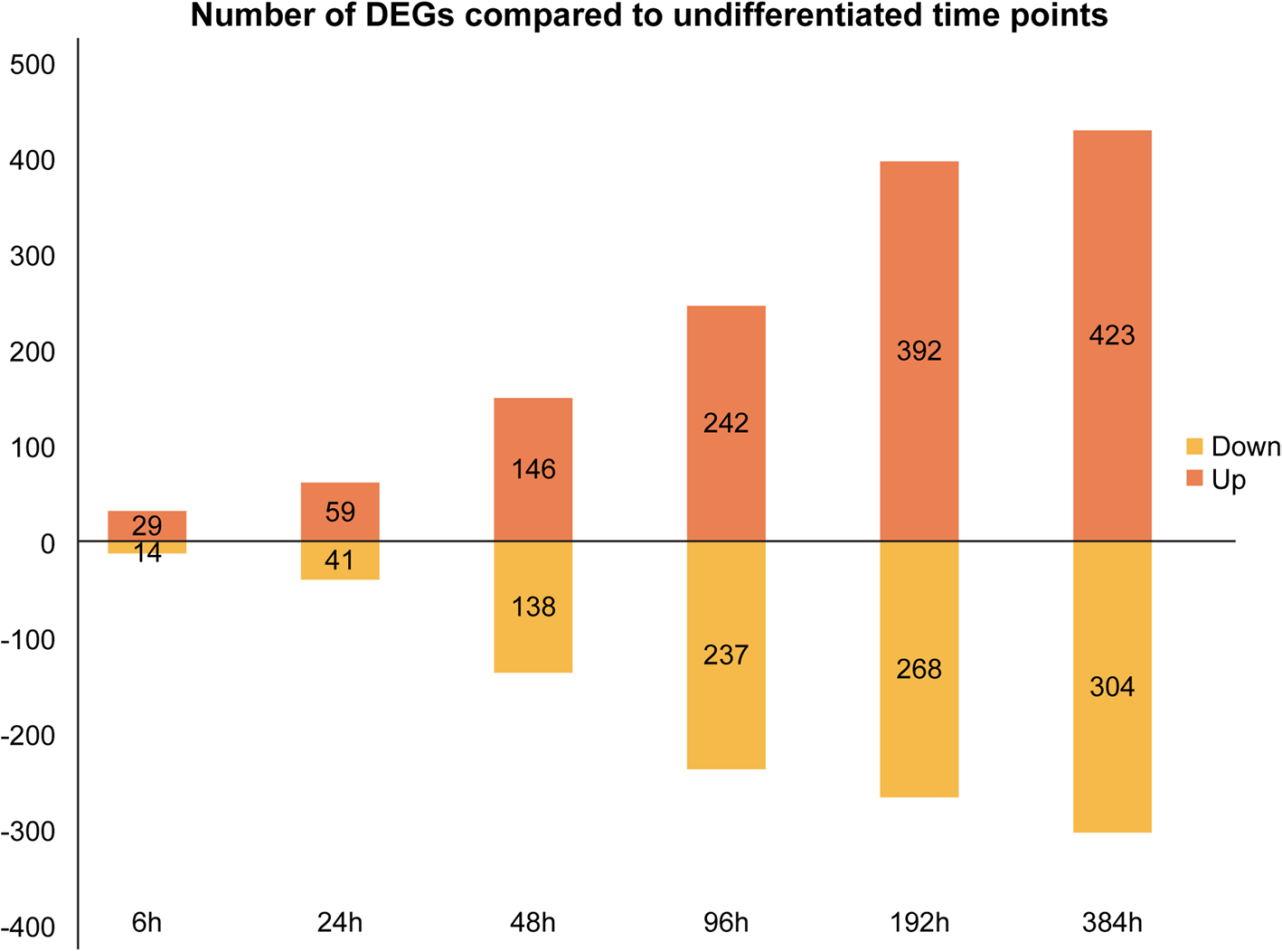
Number of differentially expressed genes (DEGs) at each time point of differentiation. DEGs, differentially expressed genes

**Fig. 3:**
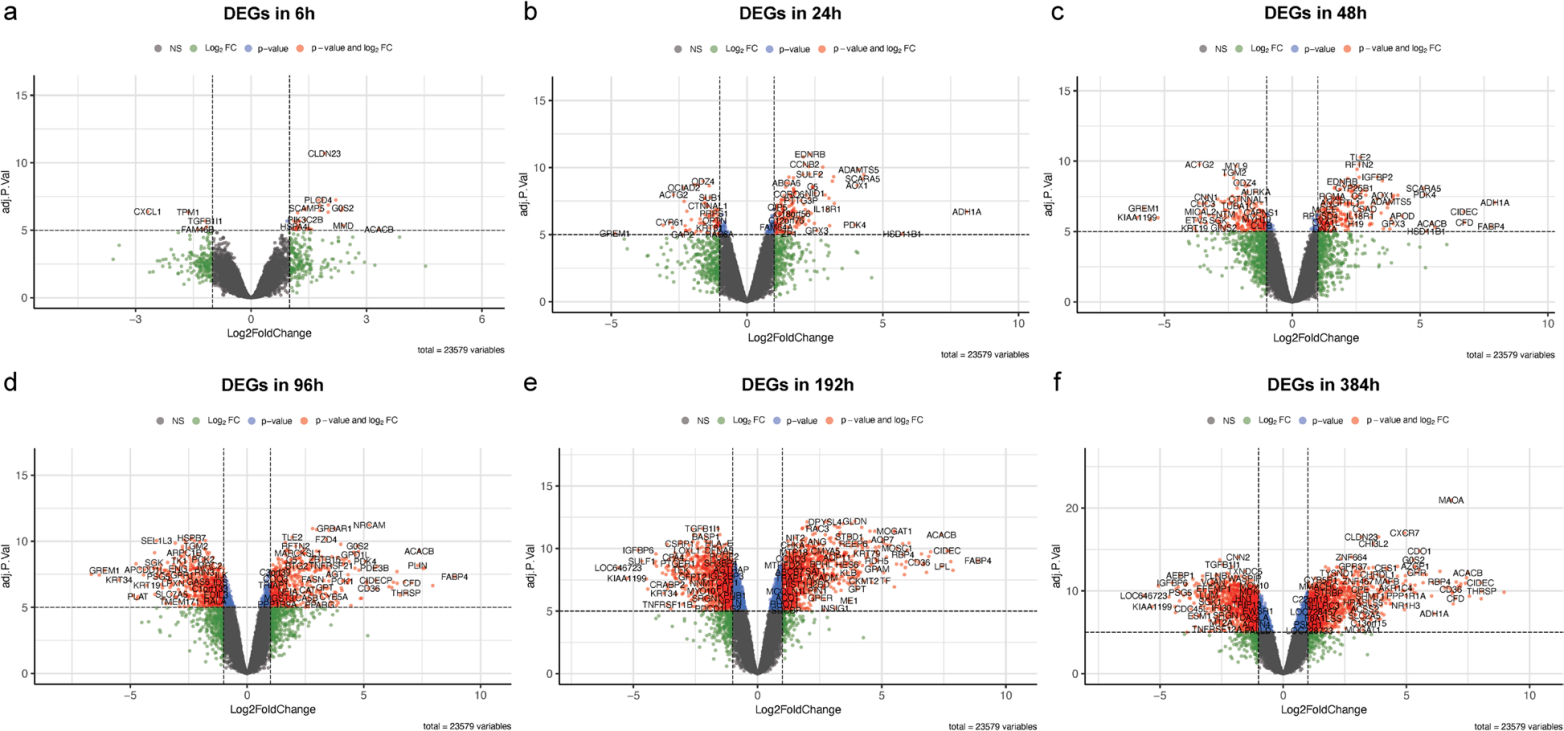
Volcano plot reflecting DEGs and their upregulation and downregulation

**Fig. 4:**
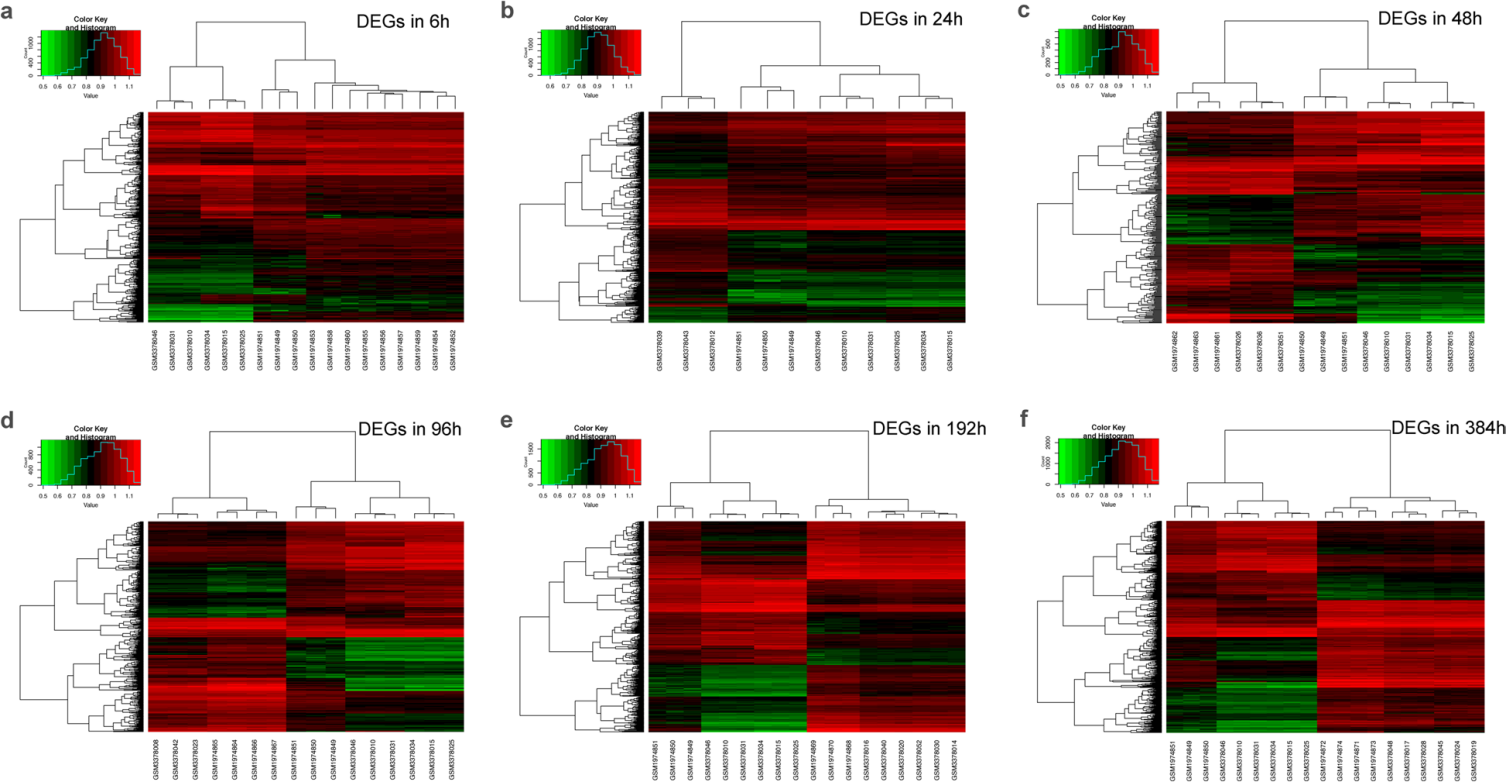
Heat map reflecting DEGs and their upregulation and downregulation

We analyzed and compared DEGs between undifferentiated samples and samples at six different stages of differentiation (Fig. 2). Volcano plots and heat maps were plotted to visualize DEGs and their upregulation and downregulation (Figs. 3, 4).

### GO and KEGG enrichment analysis

#### GO enrichment analysis

##### Biological processes

**Fig. 5:**
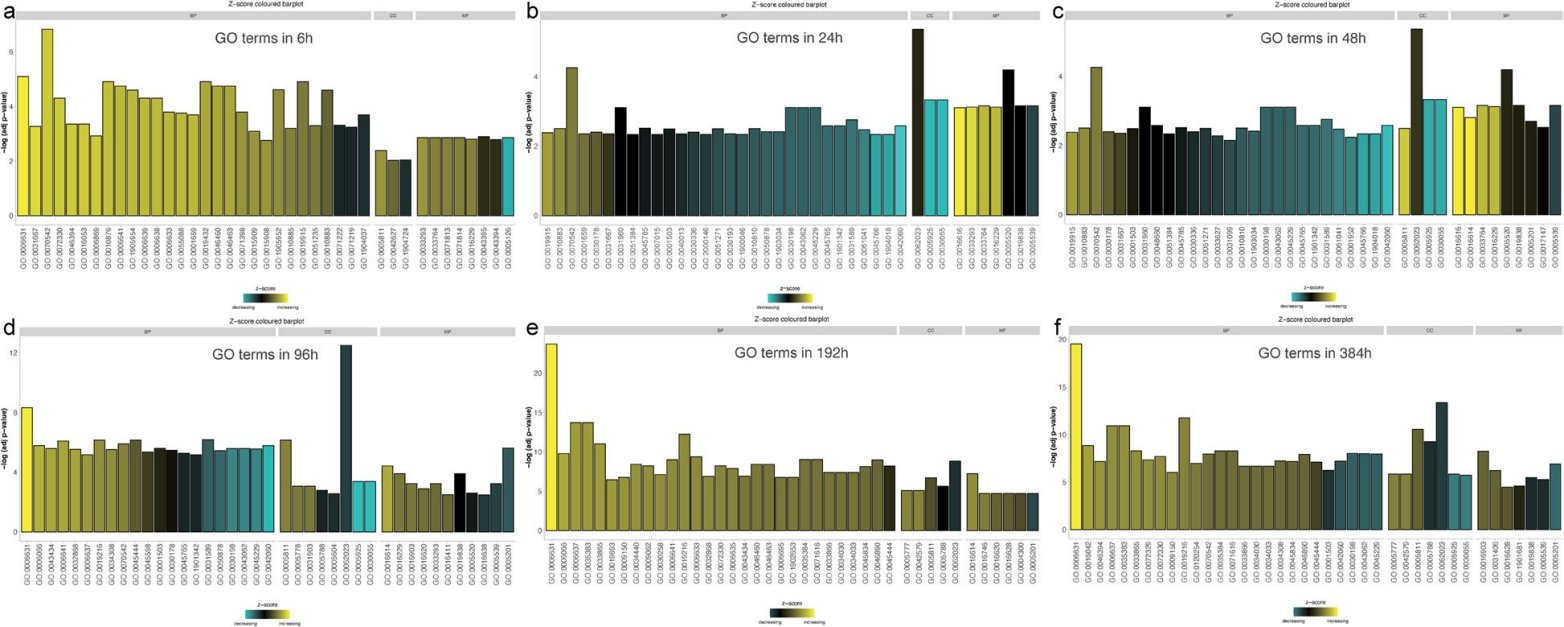
GO enrichment significance of DEGs in three functional groups at six differentiation time points. These time points represented in the histogram include molecular function (MF), biological process (BP), and cellular composition (CC). GO, gene ontology; DEGs, differentially expressed genes

**Fig. 6:**
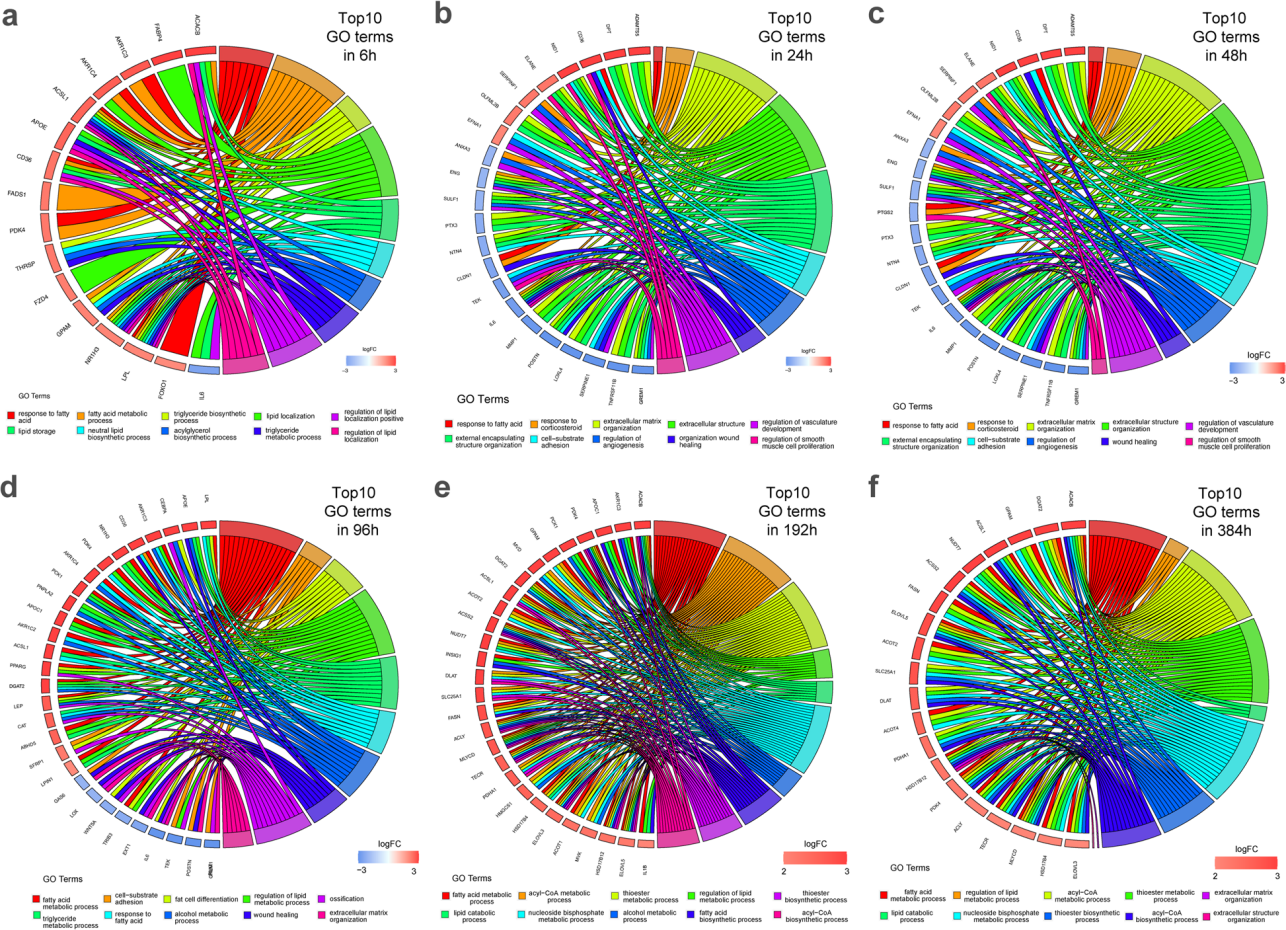
Distribution of DEGs across different GO enrichment functions at six differentiation time points. GO, gene ontology; DEGs, differentially expressed genes

The results showed (Figs. 5, 6) that fatty acid metabolism and mitochondria-related biological processes were enriched in numerous upregulated genes at any time after initiation of differentiation. Many downregulated genes were enriched in the processes of wound healing, extracellular matrix (ECM) synthesis, cell–matrix adhesion, angiogenesis, and development. Negative regulation of the Wnt signaling pathway, response to nutrient levels, and lipid localization and storage were significantly upregulated in the first 48 h. At 96 h, the fat cell differentiation, response to insulin, and neutral lipid biosynthetic process of cells became upregulated. After 192 h of differentiation, mitochondrial metabolic pathways, such as acyl-CoA metabolic process and fatty acid β-oxidation, synthesis and metabolism of cholesterol and steroids, and biological processes of nucleotide synthesis and metabolism were significantly upregulated. At 384 h, adipocyte differentiation-related pathways were further upregulated, while ossification was markedly downregulated.

##### Cellular components

The results showed that cellular components, such as lipid droplets, microbodies, and peroxisomes, were upregulated at any given time after initiation of differentiation, including cell–substrate junctions (such as basement membrane, focal adhesion, collagen-containing ECM), and other cellular components were significantly downregulated (Figs. 5, 6).

##### Molecular function

The results showed (Figs. 5, 6) that at any given time, significant upregulation of steroid dehydrogenase activity, lipoprotein particle binding, and other molecular functions were observed. However, significant downregulation of proteoglycan binding, glycosaminoglycan binding, and cytokine receptor binding was observed. After 24 h, oxidoreductase activity was significantly upregulated, whereas insulin-like growth factor binding function was downregulated.

#### KEGG enrichment analysis

**Fig. 7:**
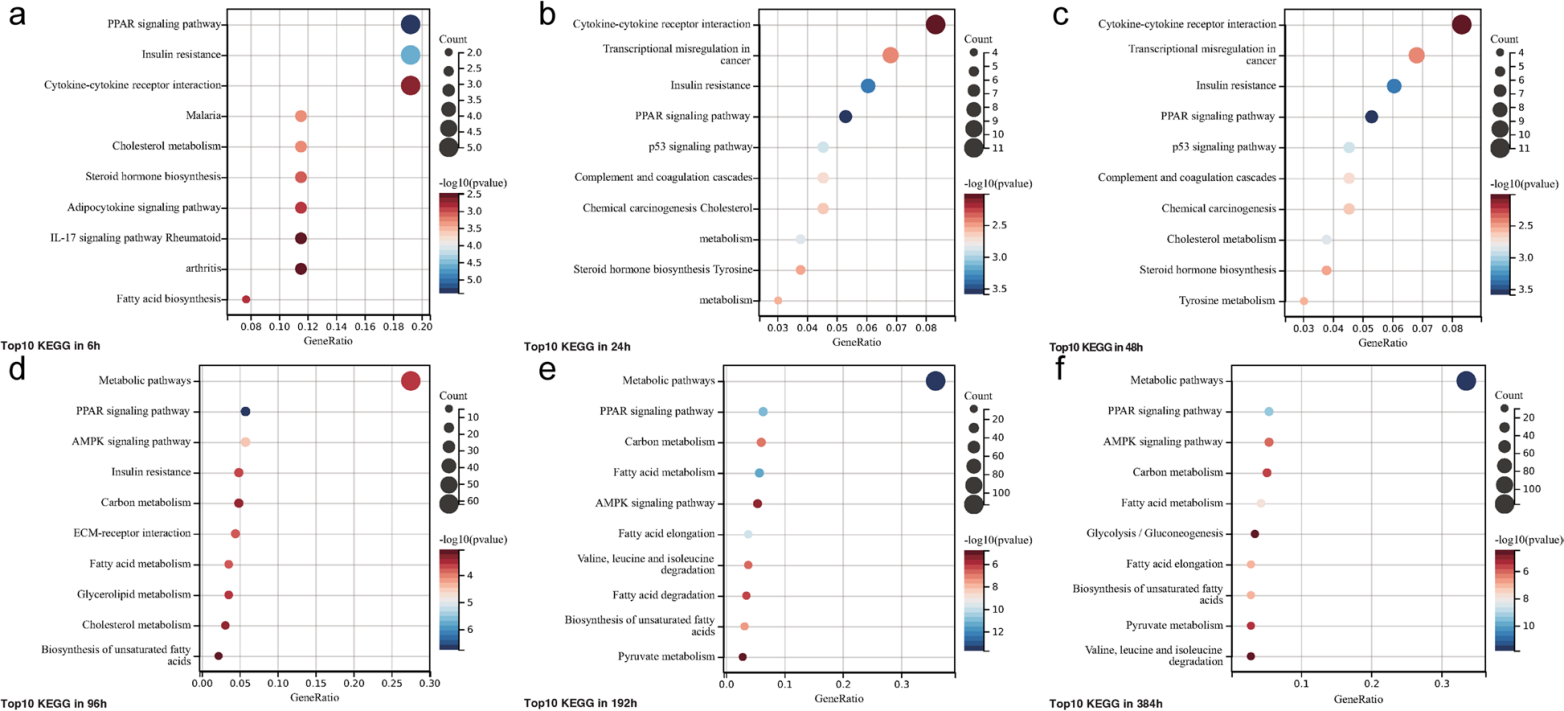
Advanced bubble chart shows enrichment of DEGs in signaling pathways at six differentiation time points. DEGs, differentially expressed genes

**Fig. 8:**
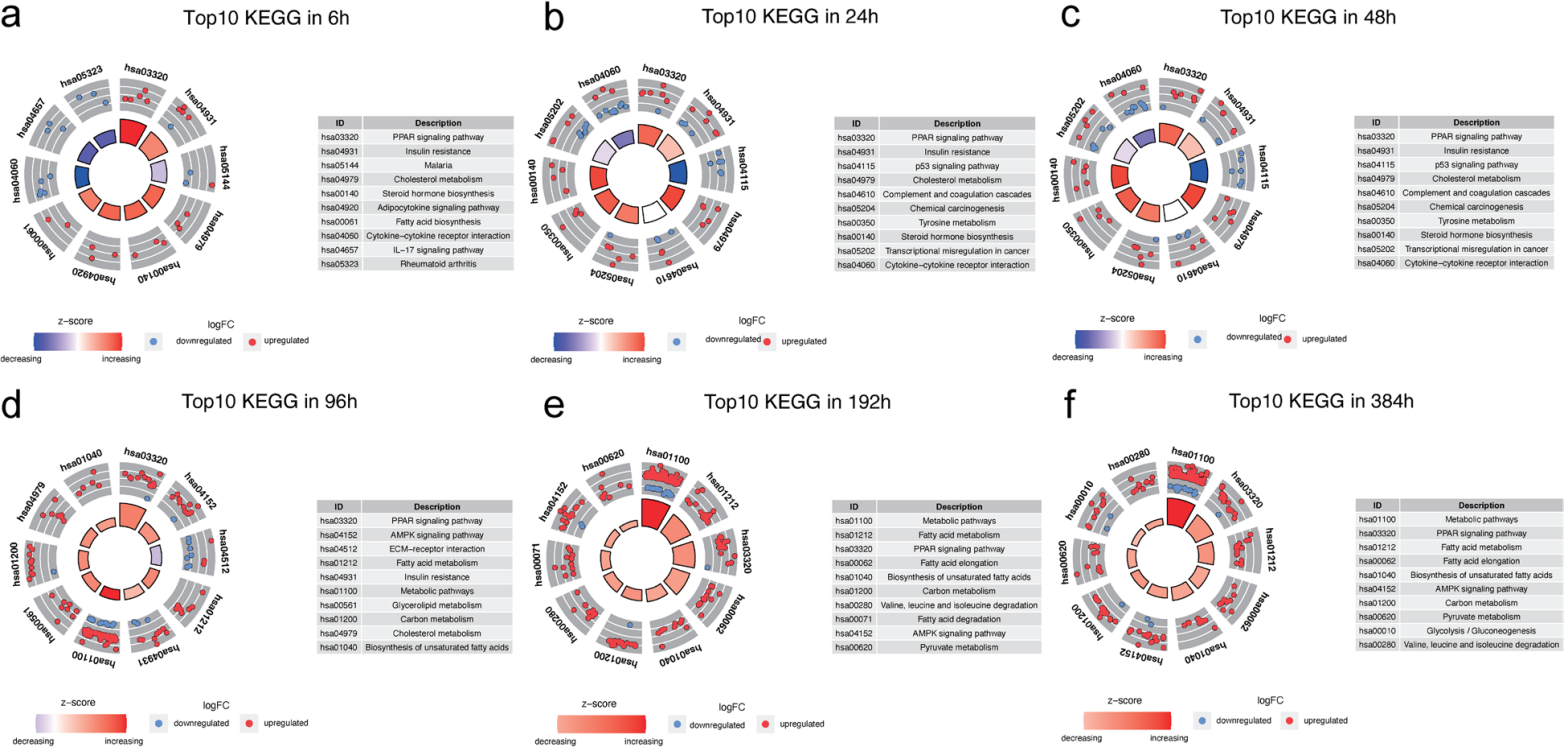
The circle diagram shows the enrichment and regulation of DEGs in the KEGG pathway. DEGs, differentially expressed genes; KEGG, Kyoto Encyclopedia of Genes and Genomes

The results of the KEGG analysis revealed (Figs. 7, 8) that numerous upregulated DEGs were significantly enriched in the peroxisome proliferator-activated receptor (PPAR) signaling pathway, insulin resistance, cholesterol metabolism, and fatty acid metabolism at any time point of differentiation. Starting at 24 h, many downregulated DEGs were enriched in the p53 signaling pathway. In addition, we observed that the enrichment of downregulated genes in the adenylate-activated protein kinase (AMPK) signaling pathway became apparent at 96 h, while a large number of upregulated genes were enriched in this pathway from 192 h. At the same time, we observed upregulation of valine, leucine, and isoleucine degradation pathway, and of the tricarboxylic acid (TCA) cycle.

### GSEA

**Fig. 9:**
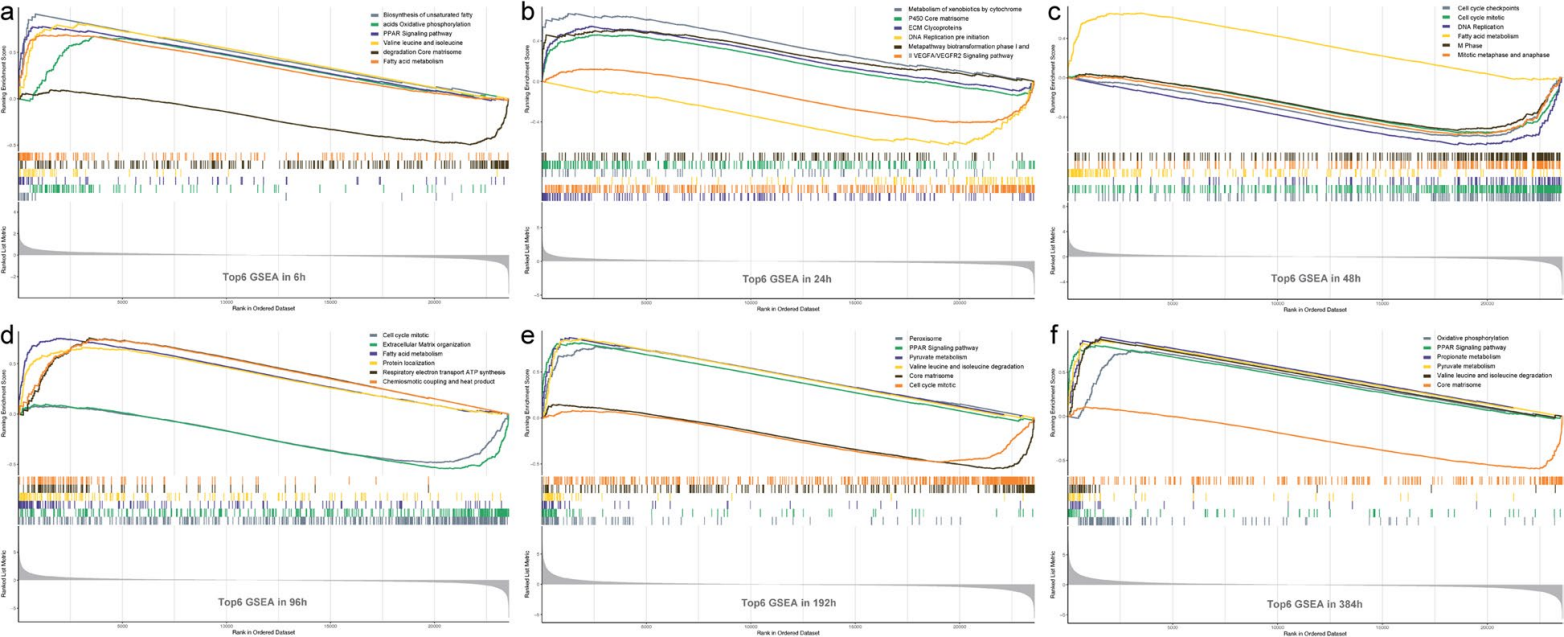
Top 6 gene sets enriched by GSEA at six differentiation time points. GSEA, Gene Set Enrichment Analysis

The GSEA revealed (Fig. 9) that significantly enriched upregulated genes were positively correlated with cell metabolism, division, and differentiation at any given time point during differentiation, including fatty acid metabolism, TCA cycle and the mitochondrial respiratory chain, the PPAR signaling pathway, and degradation of various amino acids (including valine, leucine, and isoleucine). We observed enrichment of downregulated genes in the core matrisome gene set from 24 h. In addition to the apparent enrichment of downregulated genes in the vascular endothelial growth factor signaling pathway (VEGFA/VEGFR2), upregulation was also observed. Genes were enriched in pre-initiation of DNA replication, synthesis and replication of DNA, and programmed cell death. At 48 h, genes were enriched in cell cycle checkpoints, mitotic metaphase and anaphase, and M phase, while enrichment of ECM glycoproteins became more evident in the downregulated genes in ECM organization at 96 h. Upregulated genes were noticeably enriched in fatty acid β-oxidation at 192 h.

### PPI network visualization

**Fig. 10:**
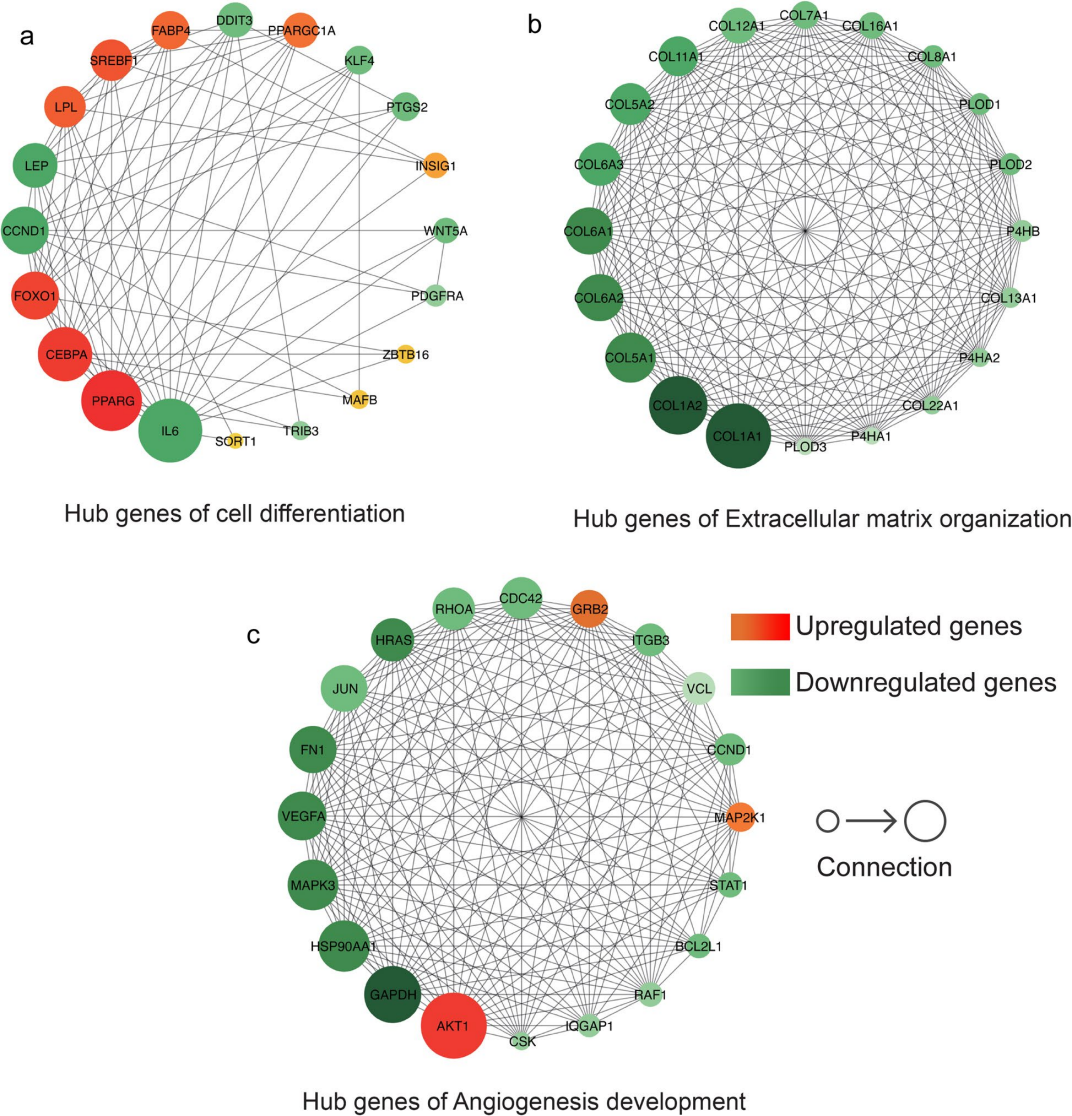
Certain hub genes in several functional pathways

Three networks, including pathways related to cell differentiation, ECM organization, and angiogenesis development, were constructed using the STRING online website. Among them, the PPI network of adipogenic differentiation-related pathways was enriched to 33 nodes and 86 edges, that of ECM-related pathways was enriched to 161 nodes and 2238 edges, and that of angiogenesis development was enriched to 184 nodes and 1532 edges. The enrichment *p*-values of the above three PPI networks were < 1.0 e^−16^, and the average local clustering coefficients were 0.626, 0.561, and 0.553, respectively. We downloaded the PPI network data and analyzed and visualized the top 20 hub genes in the three PPI networks using cytoHubba, as shown in Fig. 10 and Table 1.

**Table 1:** Regulation of certain hub genes in several functional pathways

Functional pathway	Regulation	Hub genes
Cell differentiation	Up	*PPARG, CEBPA, FOXO1, LEP, LPL, SREBF1, FABP4, PPARGC1A, INSIG1, ZBTB16, MAFB,* and *SORT1*
	Down	*IL6, CCND1, LEP, DDIT3, KLF4, PTGS2, WNT5A, PDGFRA,* and *TRIB3*
Extracellular matrix organization	Up	-
	Down	*COL1A1, COL1A2, COL5A1, COL6A2, COL6A1, COL6A3, COL5A2, COL11A1, COL12A1, COL7A1, COL16A1, COL8A1, PLOD1, PLOD2, P4HB, COL13A1, P4HA2, COL22A1, P4HA1,* and *PLOD3*
Angiogenesis development	Up	*AKT1, GRB2,* and *MAP2K1*
	Down	*GAPDH, HSP90AA1, MAPK3, VEGFA, FN1, JUN, HRAS, RHOA, CDC42, ITGB3, VCL, CCND1, STAT1, BCL2L1, RAF1, IQGAP1,* and *CSK*

### Identification of potential drugs

**Fig. 11:**
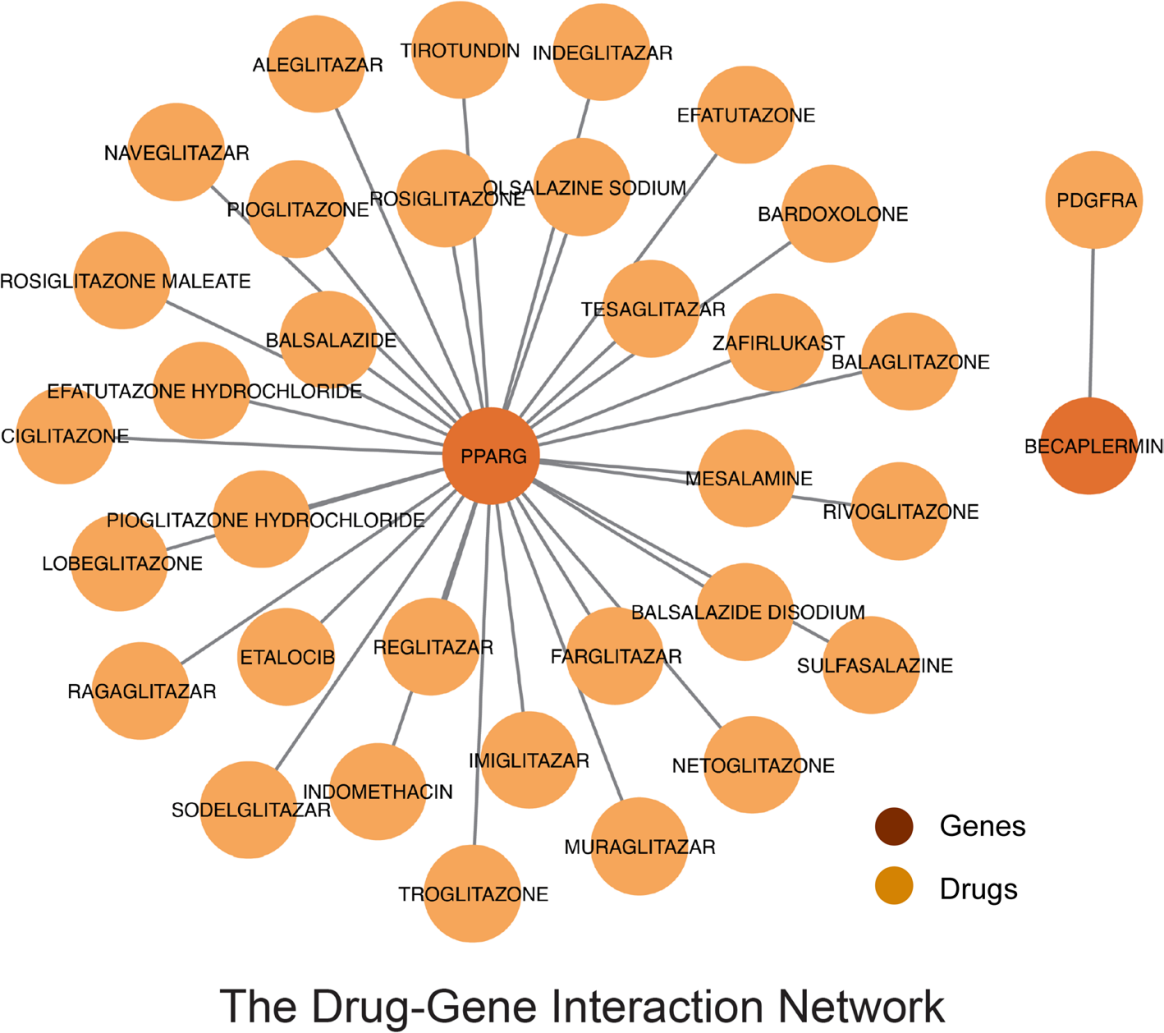
The Drug–Gene Interaction (DGI) network

Given the PPI network identification results described above, we used the Drug–Gene Interaction Database **(**DGIdb) to identify molecular compounds that can reverse or enhance these hub genes during adipogenic differentiation, which in turn allowed us to identify potential drugs that can promote angiogenesis and extracellular matrix remodeling. As shown by the DGI network, becaplermin positively regulates platelet-derived growth factor-receptor-alpha (*PDGFRA*), while 33 drugs, including netoglitazone and ragaglitazar, can positively regulate *PPARγ* as shown in Fig. 11.

## Discussion

The high absorption and low retention rates of fat grafts have substantially hindered the development of adipose tissue engineering. Differentiation and revascularization of preadipocytes in the recipient area and remodeling of ECM tissue are the main factors affecting the graft survival rate. However, the mechanisms underlying the survival and differentiation of preadipocytes in the recipient area after transplantation and their influence on the tissue in the recipient area require further study. In the present study, based on two GEO datasets, an integrated bioinformatics approach facilitated the analysis of changes in the expression of central genes to reveal changes in preadipocyte differentiation at different time points, including adipogenic differentiation, ECM and angiogenesis-related pathways, and potential biomarkers and molecular mechanisms.

We identified upregulated and downregulated DEGs at six time points of differentiation and further conducted enrichment analyses to clarify the functions of each DEG at different time points of differentiation.

Enrichment analysis results revealed that cell responses to fatty acids, anabolism of fatty acids and lipids, biological processes related to the mitochondrial respiratory chain, PPARγ signaling pathways, and AMPK signaling pathways were significantly upregulated at any given time during differentiation. The metabolism of fatty acids and lipids precedes the synthesis process. The PPARγ and AMPK signaling pathways are crucial during precursor cell differentiation. The PPAR pathway is not only involved in inflammation, adiponectin signal transduction, insulin resistance, glucose metabolism, and other cellular energy functions but is also involved in the regulation of cell proliferation, migration, and differentiation [14–16]. The AMPK signaling pathway regulates protein, lipid, and carbohydrate metabolism, autophagy, and mitochondrial homeostasis and plays an important role in regulating the energy input and output of cells [17–19]. However, ECM organization and adhesion, angiogenesis and development, wound healing, and coagulation-related biological processes were all downregulated. The adipogenic differentiation of cells appeared significantly upregulated at 96 h, and the enrichment of upregulated genes reached a peak at 384 h. Correspondingly, the anabolic processes involving various nucleotides enriched many upregulated genes at 192 h. In addition, there was a significant enrichment of downregulated genes in the p53 signaling pathway, which participates in many cellular functions depending on cell type, differentiation state, stress conditions, and environmental signals. Cell cycle arrest and apoptosis also inhibit stem cell pluripotency and cellular plasticity [20–22].

The PPI network and cytoHubba provide us with hub genes for three biological processes that affect cell survival during precursor cell differentiation. The proteins encoded by the upregulated hub genes involved in adipogenic differentiation include PPARγ, which controls the β-oxidation pathway of fatty acids by binding to the nuclear receptors of peroxisome proliferators. These activated nuclear receptors can bind to DNA-specific PPAR response elements and regulate the transcription of their target genes, indicating that they regulate adipocyte differentiation and glucose homeostasis [23–27].

CCAAT/enhancer-binding protein alpha (*CEBPA*) can directly bind to the consensus sequence 5′-T[TG]NNGNAA[TG]-3′ and, by acting as an activator of various target genes, participates in the coordination of cell proliferation arrest and stem cell differentiation [27–29]. Current studies suggest that the transcriptional activities of *PPARγ* and *CEBPA* are pre-conditions for adipocyte differentiation. Recent studies have shown that Forkhead box-O1 (*FOXO1*) is also involved in adipocyte differentiation. *FOXO1* is a target of AKT and exhibits periodic activation along the cell cycle: phosphorylation and dephosphorylation. The activated state of *FOXO1* inhibits the transcriptional activity of *PPARγ* and *CEBPA*, thereby inhibiting the adipogenic differentiation of preadipocytes [30–32]. The protein encoded by insulin-inducible gene-1 (*INSIG1*) binds to the sterol-sensing domains of two proteins, sterol regulatory element-binding protein and HMG-CoA reductase, and mediates the transport of these two proteins, thereby regulating cholesterol metabolism, lipogenesis, and glucose homeostasis [33–36].

Interleukin-6 (IL-6) is a multifunctional cytokine that regulates the immune system and metabolism. IL-6 further regulates adipocyte proliferation by activating AKT/STAT3 signaling [37] through "trans-signaling" and by synergizing with IL-1β and tumor necrosis factor. Furthermore, IL-6 induces VEGF production, which in turn leads to an increase in angiogenic activity and vascular permeability [38, 39]. DNA damage-inducible transcript 3 protein (*DDIT3*) is a multifunctional transcription factor in the endoplasmic reticulum stress response that is involved in various cellular stress responses and induces cell cycle arrest and apoptosis [40, 41]. Krüppel-like factor 4 (*KLF4*) transcription factor can be simultaneously used as an activator or inhibitor. KLF4 can activate its own transcription by binding to the promoter regions of its own genes. Experiments have shown that, as an activator, it can maintain the undifferentiated state of stem cells and inhibit differentiation [23, 42]. *PDGFRA*, which can promote or inhibit cell proliferation and migration, has been shown to play an important role in the differentiation of bone marrow-derived mesenchymal stem cells [43, 44].

ECM tissue-related hub genes are downregulated at all time points after initiation of differentiation. These genes mainly include the collagen family (*COL*), such as collagen type I (*COL1A1/COL1A2*), one of the main components of the ECM, which not only binds various cellular components but also participates in the assembly of type V collagen, a key determinant of tissue-specific matrix [45]. Recent studies suggest that collagen VI and its cleavage products may be involved in fat remodeling [46]. Collagen type VII, the basement membrane protein of stratified squamous epithelium, forms anchoring fibrils by interacting with collagen type IV in the ECM, which in turn promotes the adhesion of the basement membrane to the tissue [47]. Collagen type VIII is the main component of the basement membrane behind the corneal endothelial cells and a component of the vascular endothelium [48–50]. Collagen types XIII [51, 52] and XVI [53] are involved in integrin-mediated recognition and adhesion between cells and regulate various functions, such as cell migration, adhesion, and morphology during tissue growth. The prolyl 4-hydroxylase subunit alpha-1/alpha-2 (*P4HA1/P4HA2*) catalyzes the post-translational modification of 4-hydroxyproline in the -Xaa-Pro-Gly- sequence in various collagens and has been shown to be an important regulator in various cancers. *PLOD1/2/3* can catalyze the formation of hydroxylysine residues in the -Xaa-Lys-Gly- sequence in collagen, which can serve as an attachment point between collagen molecules and improve stability, and has recently been recognized as an important biomarker for cancer prognosis [54, 55].

Proteins encoded by upregulated hub genes associated with the development of angiogenesis, such as AKT kinase, can regulate many biological processes, including metabolism, proliferation, and angiogenesis, by mediating the phosphorylation of serine/threonine in its various downstream substrates. More than 100 candidate substrates have been reported to date, but no specificity has been reported for most of them [56–58]. Downregulated hub genes are closely related to angiogenesis development and ECM adhesion, including vascular endothelial growth factor-A (*VEGFA*) [59], which encodes a protein that can induce proliferation and migration of vascular endothelial cells, inhibit cell apoptosis, and induce vascular permeability while functioning as an indispensable cytokine that promotes angiogenesis [60–62]. The protein encoded by mitogen-activated protein kinase 3 (*MAPK3*) is an extracellular signal-regulated kinase that is involved in cell signaling cascades and responds to various extracellular signals by regulating cell proliferation, differentiation, and cell cycle [63–65]. The protein encoded by integrin subunit beta 3 (*ITGB3*) is involved in the assembly of integrins, which play important roles in cell adhesion and cell surface-mediated signaling [66]. In addition, fibronectin 1 (*FN1*) and genes encoding Ras proteins were downregulated.

Overall, the upregulated central genes showed a positive regulatory effect on adipogenic differentiation, providing support for the "graft replacement theory" at the transcriptome and proteome levels, while the downregulated central genes were concentrated in blood vessels, formation and development of ECM tissue, and the adhesion between cells and tissues. This is consistent with the clinical situation, where it is difficult to establish tissue and blood supply connections between the recipient area and donor fat after fat transplantation.

Further, we investigated the drugs or molecular compounds that may interact with the above-mentioned hub genes using the DGIdb database, hoping to identify potential drugs or molecular compounds that may improve the graft survival rate after fat transplantation. *PDGFRA* can promote or inhibit cell proliferation and cell migration and has been shown to play an important role in the differentiation of bone marrow mesenchymal stem cells. Becaplermin, as an agonist of PDGFRA that increases fibroblasts by increasing cell proliferation, cell migration, and ECM deposition, accelerates wound healing and promotes the formation of granulation tissue and blood vessels, and the synthesis of ECM [67, 68]. The protein encoded by *PPARG* is PPARγ, a key regulator of adipocyte differentiation [69]. Studies have shown that PPARγ agonists, such as netoglitazone and ciglitazone, can effectively induce bone marrow adipocyte formation and induce changes in the weight of extramedullary fat depots [70].

There are several limitations to our study. The two datasets were only differentiated for a maximum of 16 days, although the differentiation of preadipocytes to maturity generally occurs over two weeks. Longer differentiation time durations may provide new biological information. Differences in transcriptome and proteome expressions before and after transplantation of recipient tissues should be studied further to elucidate the exact mechanisms. There is no relevant research regarding drug–gene interactions and druggable genome for most of the identified hub genes. Nonetheless, the identification of these hub genes provides directions for future studies; promoting upregulation of hub genes involved in the positive regulation of adipogenic differentiation, revascularization, and ECM synthesis or reversing downregulation of hub genes involved in the negative regulation can promote establishment of the connection between the fat graft and recipient tissue, thereby improving long-term survival of fat graft.

## Conclusions

The challenge posed by fat transplantation lies in its low long-term survival rate. Preadipocytes cannot only increase the number of adipocytes remaining in the graft through adipogenic differentiation, but more importantly, they can secrete various cytokines to promote blood vessel proliferation. It is difficult to establish an effective blood supply and stromal connection between the graft and recipient area, which leads to the graft’s low survival rate, and the underlying biological mechanism is still unclear. To this end, our work provides potential targets for improving the long-term survival of fat grafts by identifying the central genes involved in cell differentiation, ECM synthesis, and angiogenesis in preadipocytes during differentiation. We also explored some drugs that interact with these central genes. However, drug–gene interactions for these compounds and the identified hub genes require further investigation.

## Methods

### Microarray data

In this study, the public gene/microarray profiling database GEO (http://www.ncbi.nlm.nih.gov/geo) was used to search and match with “Adipocytes,” “SGBS,” and “Homo sapiens” as keywords. The inclusion criteria were as follows: (i) SGBS preadipocytes differentiated and cultured for different time periods were used as the experimental group, and (ii) SGBS preadipocytes cultured without differentiation were used as the control group. We extracted the gene chips GSE76131 [71] and GSE119593 [72] from the GEO database. Both groups of microarray platforms used were the GPL10558 Illumina HumanHT-12 V4.0 expression beadchip [73]. GSE76131 includes transcriptome datasets for in vitro-induced differentiation, including six time points (0, 6, 48, 96, 192, and 384 h) with 3–9 replicates per time point, for a total of 26 sample files [71]. GSE119593 includes transcriptome data collected at six time points (0, 24, 48, 96, 192, and 384 h) during in vitro induction of SGBS differentiation, with 3–6 replicates per time point, with a total of 46 sample files [72]; excluding the experimental group using fructose medium and retaining only the ordinary differentiation medium group at different time periods left a total of 27 sample files. The raw data and platform information files from GSE76131 and GSE119593 were downloaded.

### Microarray data merging and normalization

The R software (version 4.1.2) (https://www.r-project.org/) was used to merge the GSE76131 and GSE119593 datasets, and the platform file was used for annotation. The ComBat function of the sva (version 3.1.5) package was used to remove the batch effect of the gene expression matrix [74], and the normalizeBetweenArrays function of the limma package (version 3.50.1) was used to normalize the gene expression matrix [75].

### Identification of DEGs

We used the limma package [75] to set |logFC|> 2 & adj.P.Val(FDR) < 0.05 as the screening criteria, and analyzed the DEGs between the undifferentiated samples and samples from six differentiation stages. The EnhancedVolcano [76] and pheatmap [77] functions were used to create volcano and heat maps, respectively, in order to visualize DEGs and their upregulation and downregulation.

### Enrichment analysis

GO analysis defines and describes the functions of genes and proteins and annotates which pathway or GO terms for each gene may be involved [78]. KEGG analysis was used to understand the functional enrichment of high-level functions and biological systems, which can reveal the enrichment of gene sets in specific pathways [79]. In this study, the clusterProfiler v3.14.0 [80] and GOplot v1.0.2 packages [81] were used to perform GO/KEGG analysis and visualization of DEGs at each time point of differentiation. The thresholds were set as *p* < 0.01 and *q*-value < 0.01 (GO), and *p* < 0.05 and *q*-value < 0.05 (KEGG).

### GSEA

All genes in the samples at each time point of differentiation were further analyzed using GSEA [82]. The gene set data were obtained from the Molecular Signatures Database (http://www.gsea-msigdb.org/) C2 Selected Gene Set; 2982 genes included in the five pathway databases were included in the CP canonical pathway gene set. DEGs were subjected to GSEA using the clusterProfiler package with 1000 genome permutations per analysis. The screening criterion was adjusted to adjusted *p-*value < 0.01.

### PPI network construction and hub gene identification

STRING [83] was used to identify and predict the DEGs involved in adipogenic differentiation, ECM, and revascularization-related pathways in GO, KEGG and GSEA, in order to construct and download the PPI network of DEGs data with minimum required interaction score > 0.4. Hub genes in the network were identified using cytoHubba [84], which is a universal and efficient plugin for Cytoscape (https://cytoscape.org/).

### Identification of potential drugs

The DGIdb provides information on drug–gene interactions and druggable genome [85]. We used DGIdb to predict potential drugs that may interact with the hub gene in the results obtained from previous research, and used Cytoscape to visualize these results.

## Data Availability

The datasets used and/or analyzed during the current study are available from the corresponding author on reasonable request.

## Abbreviations

ECM: Extracellular matrix
DEGs: Differentially expressed genes
GSEA: Gene Set Enrichment Analysis
PPI: Protein–protein interaction
SGBS: Simpson–Golabi–Behmel syndrome
GEO: Gene Expression Omnibus
GO: Gene Ontology
KEGG: Kyoto Encyclopedia of Genes and Genomes
PPAR: Peroxisome proliferator-activated receptor
AMPK: Adenylate-activated protein kinase
TCA: Tricarboxylic acid
VEGF: Vascular endothelial growth factor
DGIdb: Drug–Gene Interaction Database
PDGFRA: Platelet-derived growth factor-receptor-alpha
CEBPA: CCAAT/enhancer-binding protein alpha
FOXO1: Forkhead box-O1
INSIG1: Insulin-inducible gene-1
IL-6: Interleukin-6
DDIT3: DNA -damage-inducible transcript -3 protein
KLF4: Krüppel-like factor 4
COL: Collagen
P4HA1/P4HA2: Prolyl 4-hydroxylase subunit alpha-1/alpha-2
MAPK3: Mitogen-activated protein kinase 3
ITGB3: Integrin subunit beta 3
FN1: Fibronectin 1
